# Supplementary material for: Bioremediation of Hexavalent Chromium by Chromium Resistant Bacteria Reduces Phytotoxicity
Source: Int J Environ Res Public Health. 2020 Aug 19;17(17):6013. doi: 10.3390/ijerph17176013 (PMC7504174; doi:10.3390/ijerph17176013)
Supplement: Supplementary file 1 [file ijerph-17-06013-s001.pdf]

Figure S1. Locations from where the 14 samples were collected.

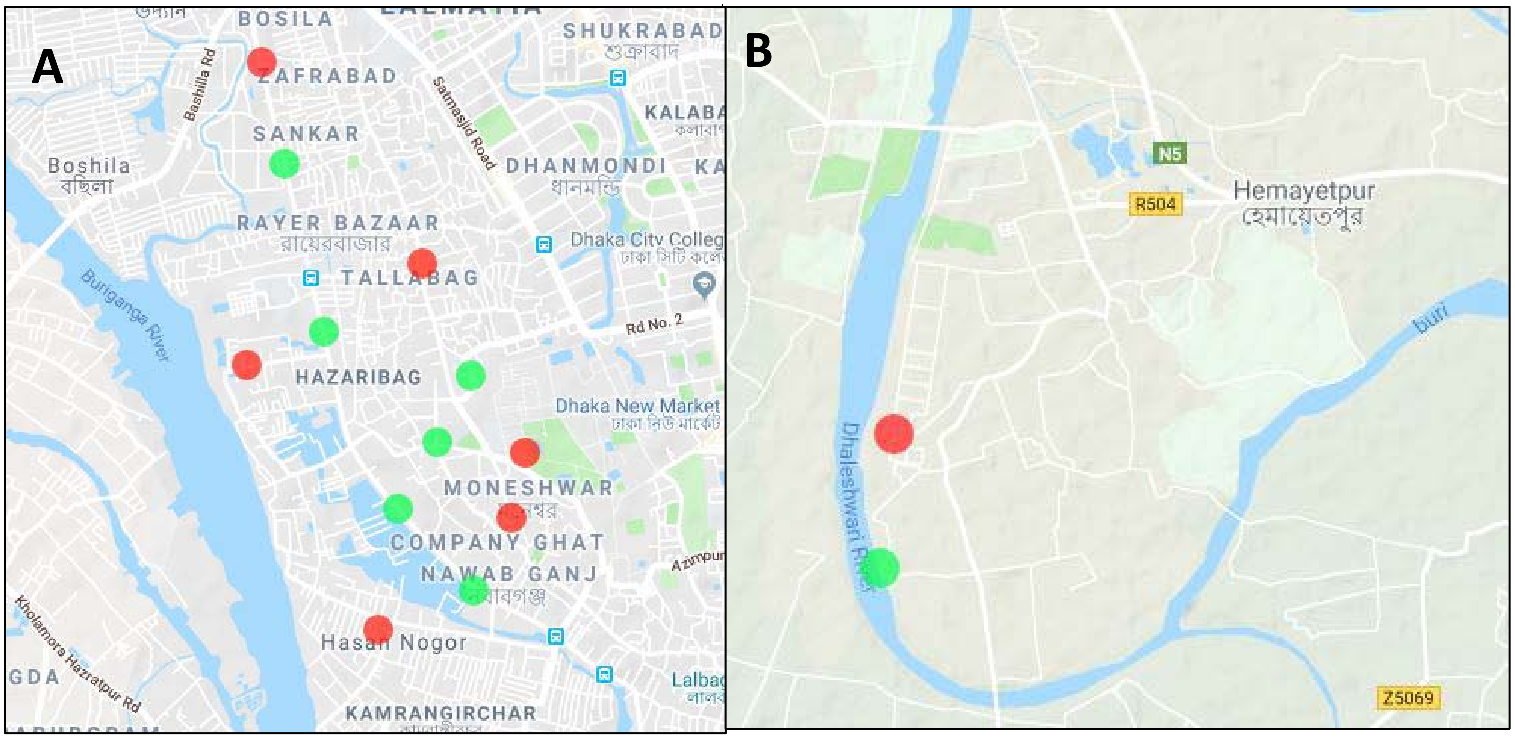

12 samples were collected from Hazaribagh area of Dhaka, Bangladesh (A) and 2 samples from Hemayetpur, Dhaka, Bangladesh (B).

Figure S2. Reduction of Cr(VI) by the isolate SH-1 in minimal salt broth with an initial concentration of 100 mg/L after 24h and 48h of incubation at 37°C.

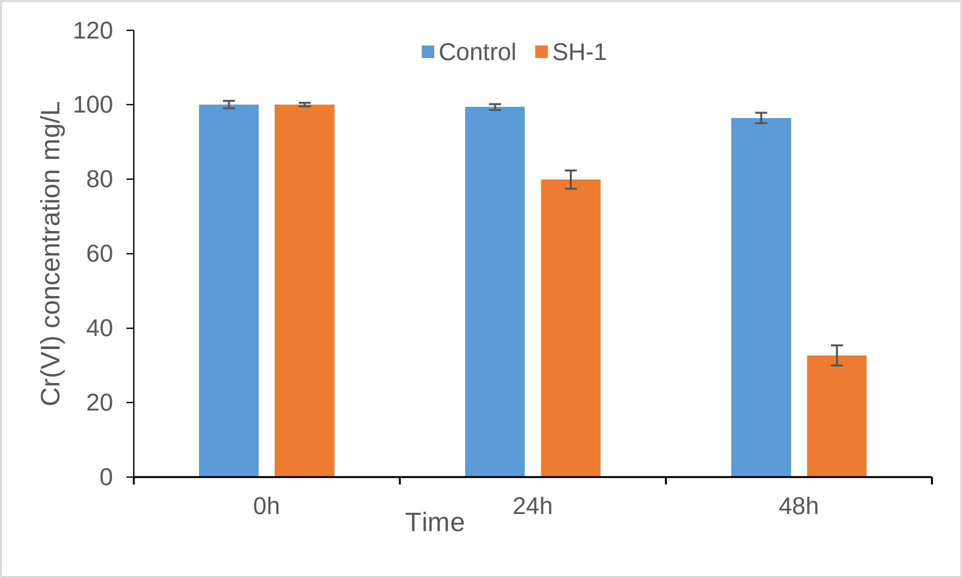

Figure S3. Pseudo-first order kinetics plots for biosorption of Cr(VI) by live and dead bacterial biomass.

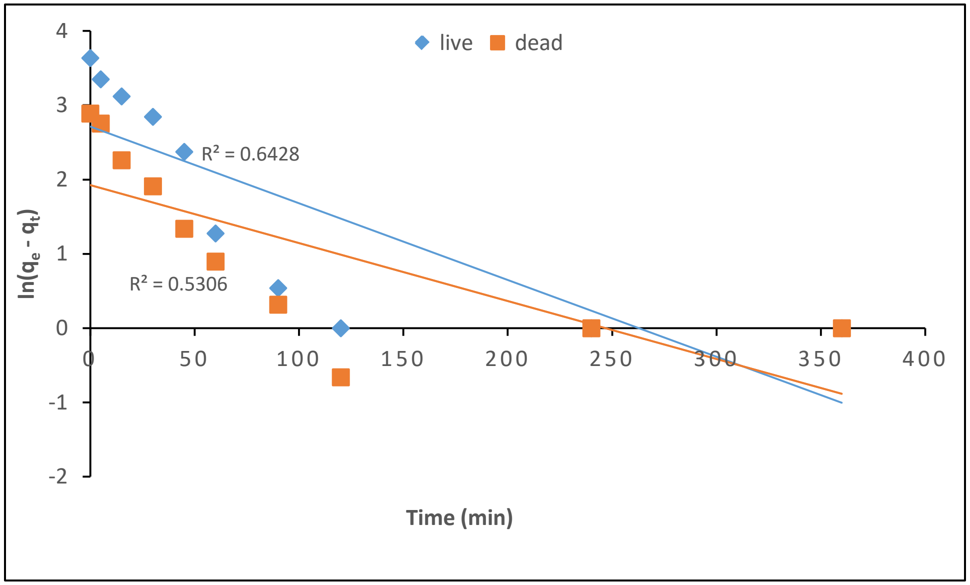

Figure S4. The EDX analysis of biomass of *Klebsiella sp.* (SH-1). (a) EDX of controlled biomass and (b) chromium treated biomass.

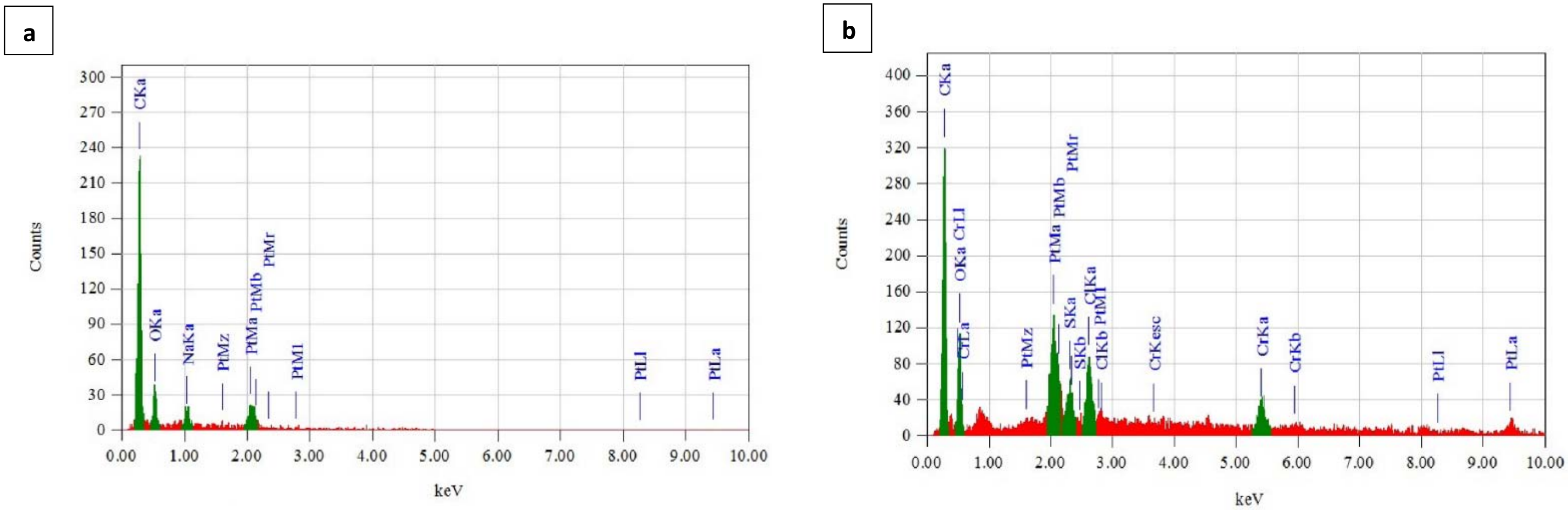

Table S1. Analysis of physicochemical properties of tannery effluents.

Serial No	Sample ID	pH	Conductivity (µs/cm)	TDS (mg/L)	Salinity (ppt)	Turbidity (NTU)	Temperature (°C)	COD (mg/L)	Chloride (mg/L)	Total Cr (mg/L)
1	S-01	6.88	752	376	0.4	91.5	24.3	405	68.86	0.009
2	S-02	7	874	437	0.4	127	23.9	436	79.84	0.005
3	S-03	8.81	2580	6290	7.2	246	23.5	1430	2619.75	4.203
4	S-04	8.61	9770	4880	5.5	215	18.4	2301	1646.7	16.921
5	S-05	7.32	7770	3850	4.3	378	24.6	1278	1546.9	5.944
6	S-06	8.64	16230	8110	9.5	367	24.7	2728	174.69	0.414
7	S-07	9.51	17760	8880	10.5	519	20.8	2553	4366.25	0.165
8	S-08	9.12	11690	5850	6.7	341	21.2	1526	1816.36	0.329
9	S-09	8.52	10430	5210	5.9	86.2	23.6	1063	1846.3	0.409
10	S-10	6.6	3920	1959	2.1	36.1	18.9	361	823.35	33.841
11	S-11	5.35	9730	4860	5.5	86.7	21.6	865	1946.1	250.303
12	S-12	3.61	106300	68000	74.5	140	26.1	7788	3500.0	1423.05
13	S-13	3.57	93100	59500	63.6	12.67	26.0	5847	4000.0	1055.41
14	S-14	8.63	16490	8250	9.7	371	20.8	2964	4091.8	0.588

Table S2. Bangladesh national standards – waste discharge quality standards for industrial units and projects: quality at discharge point.

Parameter	Unit	Location of Final Disposal		
		Inland Surface Water[1]	Public Sewer[1]	Irrigated Land[1]
Cadmium (Cd)	mg/L	0.05	0.5	0.5
Chloride (Cl-)	mg/L	600	600	600
Chromium (hexavalent Cr)	mg/L	0.1	1.0	1.0
Chromium (total Cr)	mg/L	0.5	1.0	1.0
COD	mg/L	200	400	400
Copper (Cu)	mg/L	0.5	3.0	3.0
Electrical Conductivity	μMho/cm	1200	1200	1200
Lead (Pb)	mg/L	0.1	0.1	0.1
Manganese (Mn)	mg/L	5	5	5
Nickel (Ni)	mg/L	1.0	1.0	1.0
pH		6-9	6-9	6-9
Temperature – Summer	^{o}C	40	40	40
Temperature – Winter	^{o}C	45	45	45
Total Dissolved Solids (TDS)	mg/L	2100	2100	2100
Zinc (Zn)	mg/L	5.0	10.0	10.0

Notes: (1) *Land Surface Water* refers to any pond, tank, water body, water hole, canal, river, spring or estuary *Public Sewer* refers to any sewer connected with fully combined processing plant including primary and secondary treatment *Irrigated Land* refers to an appropriately irrigated plantation area of specified crops based on quantity and quality of wastewater

Source: http://old.doe.gov.bd/publication_images/15_etp_assessment_guide.pdf

Table S3. Morphological characteristics of Cr (VI) resistant isolates from tannery effluents.

Serial No.	Sampl e ID	Isolate ID	Shape	Margin	Elevation	Size	Texture	Apperance	Pigmentation	Optical property
1	S-03	HB1	Circular	Entire	Flat	Moderate	Smooth	Glistenning	White	Translucent
2	S-03	HB2	Circular	Entire	Flat	Small	Smooth	Glistenning	White	Transparent
3	S-04	HB3	Circular	Entire	Raised	Moderate	Smooth	Glistenning	Yellow	Translucent
4	S-04	HB4	Circular	Entire	Flat	Moderate	Smooth	Glistenning	White	Translucent
5	S-04	HB5	Circular	Undulate	Raised	Small	Rough	Dull	Greyish	Opaque
6	S-05	HB6	Circular	Entire	Convex	Punctiform	Smooth	Glistenning	White	Transparent
7	S-06	HB7	Irregular	Entire	Flat	Moderate	Smooth	Dull	Greyish	Opaque
8	S-06	HB8	Circular	Entire	Convex	Large	Smooth	Glistenning	Yellow	Translucent
9	S-08	HB9	Circular	Curled	Raised	Moderate	Smooth	Glistenning	White	Transparent
10	S-09	HB10	Circular	Entire	Raised	Small	Smooth	Glistenning	white	Translucent
11	S-09	HB11	Circular	Entire	Convex	Moderate	Smooth	Glistenning	Yellow	Transparent
12	S-10	HB12	Spindle	Curled	Flat	Moderate	Rough	Dull	Greyish	Opaque
13	S-10	HB13	Circular	Entire	Flat	Small	Smooth	Glistenning	white	Transparent
14	S-10	SH2	Circular	Entire	Raised	Moderate	Smooth	Glistenning	Yellow	Transparent
15	S-10	HB15	Circular	Entire	Raised	Moderate	Smooth	Glistenning	Yellow	Translucent
16	S-11	HB16	Circular	Curled	Convex	Large	Powdery	Glistenning	Greyish	Opaque
17	S-11	HB17	Pin-point	Entire	Raised	Small	Smooth	Glistenning	white	Translucent
**18**	**S-11**	**SH1**	**Circular**	**Entire**	**Raised**	**Small**	**Smooth**	**Glistenning**	**White**	**Transparent**
19	S-11	HB19	Irregular	Curled	Convex	Moderate	Rough	Dull	Greyish	Opaque
20	S-11	HB20	Circular	Entire	Raised	Small	Smooth	Glistenning	white	Transparent
21	S-14	HB21	Pin-point	Entire	Pulvinate	Small	Smooth	Glistenning	Yellow	Translucent
22	S-14	HB22	Circular	Curled	Convex	Large	Smooth	Glistenning	white	Transparent
23	S-14	HB23	Pin-point	Entire	Convex	Small	Smooth	Glistenning	Greyish	Transparent
24	S-14	HB24	Irregular	Curled	Pulvinate	Large	Smooth	Dull	Greyish	Opaque
25	S-14	HB25	Pin-point	Entire	Raised	Small	Smooth	Glistenning	Greyish	Transparent
26	S-14	HB26	Irregular	Entire	Convex	Moderate	Smooth	Dull	Greyish	Translucent
27	S-14	HB27	Circular	Entire	Convex	Large	Smooth	Glistenning	White	Transparent
28	S-14	HB28	Circular	Curled	Pulvinate	Large	Rough	Glistenning	Yellow	Translucent

Bold line indicates selected isolate for further study. Selected isolate was renamed as SH-1 as indicated in the Table S4.

Table S4. Antibiotics sensitivity of SH-1.

Antimicrobial	MIC	Interpretation	Antimicrobial	MIC	Interpretation
Amikacin	<=2	S	Ertapenem	<=0.5	S
Gentamicin	<=1	S	Imipenem	<=0.25	S
Amoxicillin/Clavulanic Acid	4	S	Meropenem	<=0.25	S
Ampicillin	16	R	Nalidixic Acid	4	S
Cefepime	<=1	S	Nitrofurantoin	32	S
Cefoperazone/Sulbactam	<=8	S	Piperacillin/Tazobactam	<=4	S
Ceftriaxon	<=1	S	Tigecycline	<=0.5	S
Cefuroxime	2	S	Trimethoprim/Sulfamethoxazole	<=20	S
Cefuroxime Axetil	2	S	Cefixime*		S
Ciprofloxacin	<=0.25	S	Ceftazidime*		S
Colistin	<=0.5	S			

‘*’ = Deduced Drug, ‘S’ = Sensitive, ‘R’= Resistant

Table S5. Pseudo-first order kinetics constants for biosorption of Cr(VI) by live and dead bacterial biomass.

Biomass	K_2 (g/mg min)	q_e cal. (mg/g)	q_e exp. (mg/g)	R^2	Experimental error (%)
Live	0.0017	40.0	38.03	0.9946	4.925
Dead	0.0045	19.23	18.2	0.9937	5.36
